# Visual effect of air pollution on the need for arousal and variety-seeking behavior

**DOI:** 10.3389/fpsyg.2024.1342267

**Published:** 2024-05-23

**Authors:** Han Zhang, Guanling Huang, Ping Lin, Xiuqi Chen, Wenhe Lin

**Affiliations:** ^1^Newhuadu Business School, Minjiang University, Fuzhou, Fujian, China; ^2^Fujian Agriculture and Forestry University, Fuzhou, Fujian, China

**Keywords:** air pollution, achromatic color, variety-seeking, need for arousal, visibility

## Abstract

Research on air pollution, one of the most common environmental factors, has primarily focused on its effects on physical, mental, and cognitive health. However, air pollution-induced achromatic color of an environment, which is a prominent feature of air pollution, has received little attention. This study explored the visual effects of air pollution on the variety-seeking purchase behavior of consumers through two scenario-based experiments and primed manipulation (Study 1 and Study 2) and one natural experiment using data from a local fruit chain store (Study 3). Study 1 tested the main effect of air pollution on the variety-seeking behavior and found that primed air pollution increased variety-seeking when consumers purchased beverages. Study 2 broadened the category and tested the mechanism, and the results indicated that primed air pollution increased the variety of purchased chocolates and demonstrated the mediating effect of the need for arousal. Study 3 tested the boundary condition and extended the external validity with actual purchases. The results revealed that severe air pollution increased the purchased SKUs by 22.9% and visibility reduced the moderation effect. This research extended the literature on the visual effect of air pollution by providing evidence of the effects of air pollution on variety-seeking behavior through the need for arousal. And, product managers could leverage the results by offering a greater variety of goods on days with air pollution to increase sales.

## 1 Introduction

The prevalence of air pollution is beyond our imagination. According to data from the WHO, in 2019, 99% of the global population lived in environments with air quality levels below the WHO standards, resulting in 6.7 million premature deaths.^1^ The problem is most critical in low- and middle-income countries, where 89% of premature deaths occur. Despite the relatively better air quality in developed countries, air pollution-related issues are prevalent in these countries as well, with 56% of the cities failing to meet the WHO standards. Not only is the current situation of air pollution concerning but the future prospects for air quality are also bleak. Data on particulate matter with diameters <2.5 μm and 10 μm (PM_2.5_ and PM_10_, respectively), which are the main components of air pollution, in 67 countries and 795 cities were compared by the WHO. The results revealed an alarming trend of an annual increase of approximately 8% in PM_2.5_ and PM_10_ in recent years.^2^ In developed countries, the situation is comparatively better yet not reassuring, as observed in the United States, where the PM_2.5_ levels increased by an average of 5.5% between 2016 and 2018 (Clay et al., 2021).

Air pollution has been investigated from the perspective of its effects on physical, mental, and cognitive health. Studies have shown that both long- and short-term exposure to air pollution can lead to various diseases and higher mortality (Brunekreef and Holgate, 2002). Specifically, adverse effects on the immune system (Glencross et al., 2020), respiratory system (Manisalidis et al., 2020), cardiovascular system (Hamanaka and Mutlu, 2018), and central nervous system (Genc et al., 2012), as well as a wide range of diseases (Brunekreef and Holgate, 2002; Genc et al., 2012; Hamanaka and Mutlu, 2018; Glencross et al., 2020; Manisalidis et al., 2020), can be attributed to air pollution. Recognizing the negative effects of air pollution on health, consumers are more likely to purchase masks (Zhang and Mu, 2018), air purifiers (Ito and Zhang, 2020), and health insurance (Chang et al., 2018) as well as migrate (Vuong et al., 2022a, 2023). In terms of mental health, air pollution can cause anxiety and depression in people (Crüts et al., 2008; Pun et al., 2017; Zhang et al., 2017; Lu et al., 2018; Xue et al., 2019). As a result, consumers are more likely to purchase hedonic products that improve their mood (Liu et al., 2022), investors tend to anticipate more pessimistic profit prospects (Dong et al., 2021), and people are prone to engage in unethical behaviors (Lu et al., 2018). In terms of cognitive health, research has indicated that air pollution decreases the fluid and crystallized cognition ability of children (Calderón-Garcidueñas et al., 2008), negatively affects the cognitive performance of adults (Chen and Schwartz, 2009), and impairs spatial learning (Fonken et al., 2011). As a result, consumers tend to make poorer financial decisions when they are exposed to air pollution (Li et al., 2017).

Although air pollution has attracted much attention, there is limited research on how the visual elements of air pollution affect people. Research has shown that air pollution decreases visibility and visual contrast as well as discolors the sky (Hyslop, 2009). However, how air pollution-induced achromatic color affects consumer behavior remains unclear. In this research, we analyzed the effects of air pollution-induced achromatic environments and the effects of air pollution on the variety-seeking behavior of individuals. We found that air pollution increases the variety-seeking behavior through the channel of the need for arousal, in which variety-seeking serves as a response to the need for arousal.

This research extends the current literature on air pollution and variety-seeking in three ways. First, it broadens the air pollution-related literature. As air pollution is a conspicuous topic, it has been researched from the perspective of its primary effects on physical, mental, and cognitive health, as well as its secondary effects. However, the impact of the achromatic environment caused by air pollution on behaviors remains unexplored. In this study, we investigated how air pollution affects behaviors through its visual effect. Second, to the best of our knowledge, we are the first to propose that air pollution increases people's need for arousal, thereby enriching the understanding of the effects of air pollution on people. Third, we conducted two laboratory experiments, followed by a natural experiment, extending the external validity, and found that air pollution increased the number of stock keeping units (SKUs) purchased by 22.9%. This result has practical implications.

In the following sections, we summarize the literature, illustrate the theoretical foundation, and propose hypotheses. Subsequently, we provide a detailed description of three experiments, two laboratory experiments and a natural experiment, conducted in this study. Finally, we discuss the study's contributions, limitations, and future research directions.

## 2 Theoretical background and hypotheses

### 2.1 Air pollution and behavior

The effects of air pollution on health have been widely investigated. Research has revealed that air pollution decreases physical health (Glencross et al., 2020; Clay et al., 2021), mental health (Pun et al., 2017; Lu et al., 2018), and cognitive ability (Calderón-Garcidueñas et al., 2008; Chen and Schwartz, 2009; Levav and Zhu, 2009; Fonken et al., 2011).

Given the impact of air pollution on health, many studies have further explored this topic. Due to the widely recognized critical physical health consequences of air pollution, individuals increasingly tend to engage in avoidance behaviors (Zivin et al., 2009; Vuong et al., 2022a, 2023), purchase masks (Zhang and Mu, 2018) and air purifiers (Ito and Zhang, 2020), and acquire health insurance (Chang et al., 2018). Evidence also suggests that investors tend to make poor stock trade (Huang et al., 2020) and are inclined to rely on intuition in stock exchanges (Li et al., 2017), indicating the impact of air pollution on cognitive ability.

Research has shown that air pollution leads to a more abstract construal level and increases preferences for desirability through visibility (Ding et al., 2021). In addition to visibility, an achromatic environment is a prominent consequence of air pollution. However, how air pollution-induced achromatic environments affect people's behavior remains to be investigated. This research broadens the literature by investigating the impact of air pollution on human behaviors from the perspective of the visual effect induced by air pollution.

### 2.2 Color and consumer behavior

Color is defined by three dimensions: hue, saturation, and lightness. Hue is the spectral wavelength composition of a color, which is close to the term “color” that we use. Saturation is the degree of purity, intensity, and richness of a color. Lightness refers to the relative darkness or brightness of a color (Wei et al., 2016; Labrecque, 2020). As a frequently encountered element, the effects of color on consumer behaviors have been widely investigated, specifically focusing on how product color affects product attribute evaluation and how environmental color affects consumer behaviors.

From the perspective of product color evaluation, research has focused on the influence of color on the weight, durability, friendliness, taste, health, and quality of products. Compared with light-colored products, dark-colored products are perceived as heavier (Walker et al., 2010) and more durable (Hagtvedt, 2020); products with light colors are perceived as more user-friendly (Hagtvedt, 2020). Food with high color saturation is more likely to be perceived as tasty and healthy as it is perceived to be fresh (Kunz et al., 2020) and a “natural color” increases consumers' willingness to pay for healthy food, as they are perceived to be authentic (Marozzo et al., 2020). Light reflection brightness increases the purchase intention for sport utility vehicles (SUVs) but not for compact cars, owing to the fact that light reflection brightness increases perceived premium design; however, cost is a more important factor for the purchase of compact cars (Kato, 2022).

This research is closely related to how environmental color affects consumer behaviors. Barli et al. (2012) found that stores with soft lighting (vs. bright lighting) had increased in-store time and red interiors decreased the in-store time. Warmer colors were more appealing to consumers and generated more positive responses with increased perceived warmth and emotional trust (Chan et al., 2023). A blue background (vs. a yellow background) increased perceived enjoyment and concentration, which increased the purchase intention and revisit intention (Aboubaker Ettis, 2017). In addition to color itself, other factors play a role. Research has shown that, for fashion-oriented stores, compared with orange interiors, blue interiors lead to more favorable evaluation, greater excitement, greater store patronage intention, and greater purchase intention (Babin et al., 2003). Anwar et al. (2020) found that cool colors with fast-tempo music increased pleasure and arousal compared with warm colors with slow-tempo music. Warm colors worked better in business-to-consumer (B2C) content, but cool colors were more appreciated in business-to-business (B2B) content (Kwon et al., 2022).

Most of the abovementioned literature related to environmental color focused on how in-store color affects consumer behaviors, while the effects of outdoor environment color have not been examined. This research investigated how air pollution-induced achromatic color of outdoor environments affects consumer behavior, thereby extending the literature on the influence of environmental color, especially out-store environmental color, on consumer behavior.

### 2.3 Variety-seeking as a response

An achromatic environment induced by air pollution increases the need for arousal in individuals. Arousal is a physiological state of alertness that is associated with a sense of stimulation and appraisal of an event (Gustafsson, 2023). According to the optimal level of arousal theory, people intentionally maintain an optimal level of arousal, and the greater the deviation between the actual arousal level and the optimal level, the greater is the need for arousal; people with a higher optimal arousal level tend to engage in arousal-seeking behaviors (Zuckerman, 1994). Research has shown that individuals with higher optimal arousal levels are more likely to engage in curiosity-motivated consumer behaviors and risk-taking behaviors, all of which are arousal-seeking behaviors (Steenkamp and Baumgartner, 1992). Therefore, people try to maintain their optimal level of arousal while attempting to remedy any deviation. Previous research has shown that achromatic color decreases the arousal level (Wilms and Oberfeld, 2018), which in turn increases the need for arousal. When air pollution exists, semitransparent white fog spreads in the environment, creating an achromatic environment, which intensifies the need for arousal.

Variety-seeking is a kind of response to fulfill the need for arousal and represents the tendency to diversify choices in decision episodes (Ratner et al., 1999). For example, when purchasing chocolate, people tend to choose one piece each of dark, white, and nutty chocolate rather than three pieces of dark chocolate, which is a typical variety-seeking behavior indicating a desire for a greater variety. Previous research has shown that variety-seeking behavior can be driven by consumers' personal factors, marketing strategy, purchase choice setting, and contextual factors. One of the motivations for consumers to seek variety in their purchases is the need for arousal. Choosing varied things can provide a stimulating and exciting experience to individuals (Etkin, 2016), which fulfills their need for arousal. Gullo et al. (2019) found that consumers tend to not engage in variety-seeking in the morning because there is a lack of need to experience arousal at that time. Similarly, Huang et al. (2019) found that people who are sleepy tend to increase variety-seeking because of the fulfilling effect of variety-seeking on the need for arousal. Therefore, we propose the following hypotheses:

*H1:* Air pollution increases variety-seeking.

*H2:* The need for arousal mediates the effect of air pollution on variety-seeking.

**Figure 1:**
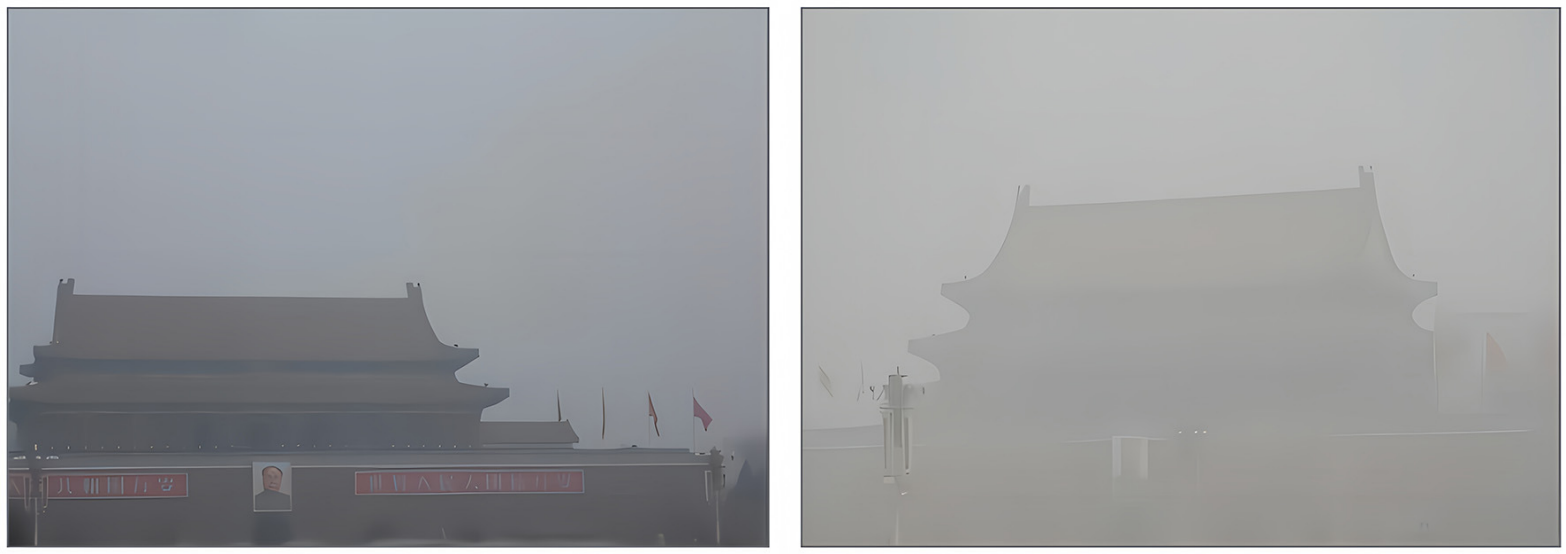
Images with almost the same environmental colors but different visibilities. The two scenes shows almost the same color. However, the scene in the left image has higher visibility which results less achromatic compared with the right one.

In the above paragraphs, we proposed that an air pollution-induced achromatic environment increases the need for arousal and that variety-seeking serves as a response. The achromatic environment plays an important role and is strongly affected by visibility. When visibility is high, achromatic fog is distracted by the surrounding environment, which decreases the effect of air pollution on variety-seeking. Although the achromatic environment and visibility are correlated, they are different constructs. The achromatic environment is about the color of the air pollution, while visibility is about transparency. The two images shown in Figure 1 share almost the same color, but the image on the left has higher visibility and is less achromatic. Overall, we hypothesize the following:

*H3:* Visibility weakens the effect of air pollution on variety-seeking.

## 3 The studies and methodology

### 3.1 Overview of the studies

In this research, we conducted three studies to examine the effects of air pollution on variety-seeking—two lab experiments (Study 1 and Study 2) and a natural experiment (Study 3). Study 1 provided the initial evidence for *H1*, namely, air pollution has a significant increasing effect on consumer's variety-seeking behavior in the presence of primed air pollution. Study 2 further supported the main effect (*H1*) and tested the mechanism of the need for arousal (*H2*). Study 3 demonstrated the external validity and tested the moderation effect of visibility (*H3*) through a natural experiment, which provides insights for managers and deepens their understanding.

### 3.2 Study 1: primed air pollution increases variety-seeking

Study 1 aimed to test the main effect (*H1*) in a scenario-based setting with primed air pollution to prove our prediction that air pollution increases consumer variety-seeking behavior.

#### 3.2.1 Participants and procedure

##### 3.2.1.1 Participants

This is a one factor (air pollution level: high air pollution vs. low air pollution) between-subjects design. A total of 110 participants were recruited for a RMB¥6.00 payment from Credamo, a well-known Chinese data collection platform. A total of 100 valid participants were recruited, with the following characteristics: 38% were men, with a mean age (M_age_) of 30.56 (standard deviation (SD) = 7.46); 78.00% were unmarried; and participants' median income was between RMB¥8,000 and ¥10,000.

##### 3.2.1.2 Air pollution manipulation

A priming methodology similar to that of Lu et al. (2018) was adopted. The participants were randomly assigned to either the high or low air pollution condition group. The subjects in the high air pollution condition group were instructed to observe six photos taken on a severely polluted day. The participants were asked, “Please imagine the air quality of the city where you are currently living, as shown in the photos, and you are breathing, working, and studying under such air quality. Then, describe how you would feel and experience living in this environment for 1 day in detail. For example, how would you feel when going outside? (minimum of 25 words).”

A similar procedure was followed for the participants in the low air pollution condition using photos taken in the same geographical locations as those of the air pollution condition but on a blue-sky day. For the manipulation check, the participants in each group were asked “How would you rate the air quality of the city on the day the photo was taken? Please rate from 1 (very bad) to 7 (very good)” (Liu et al., 2022).

##### 3.2.1.3 Variety-seeking measurement

Subsequently, variety-seeking was measured by asking participants to choose three beverages they preferred out of six different beverages (i.e., Coca-Cola, Sprite, Fanta, Mizone, Nongfu Spring, and Ice Tea). The options were randomly displayed to eliminate any sequence effect. The participants could choose zero to three bottles of each flavor, maintaining the total number of beverages as three. For example, the participant could choose three bottles of Coca-Cola or one bottle of Sprite and two bottles of Nongfu Spring, both cases totaling to three bottles. The number of chosen flavors was the measure of variety-seeking.

Several confounding factors, including participants' mood and demographic information, were captured. Participants were asked to rate their mood on a seven-point scale ranging from 1 (indicating very bad) to 7 (indicating very good) (Ding et al., 2021).

#### 3.2.2 Results

##### 3.2.2.1 Manipulation check

We used a t-test to analyze manipulation. The participants rated the air quality, which reversely measured air pollution level, and the subjects in the high air pollution condition reported a lower air quality level (M_low air pollution_ = 6.72 vs. M_high air pollution_= 1.48, *t* = 13.15, *p* < 0.001). This result indicates successful manipulation.

##### 3.2.2.2 Main effect of air pollution on variety-seeking

A linear regression model was used to test the effect of air pollution on variety-seeking. We regressed variety-seeking on air pollution and other controls, including age, income level, education level, and mood. The results indicate that air pollution increased variety-seeking significantly. On average, the participants who were provided photos of the air pollution condition chose 0.59 more categories than those who were provided photos of the blue-sky condition (*b* = 0.59, *t* = 2.70, *p* = 0.008). This result supports *H1*.

#### 3.2.3 Discussion

We conducted a scenario-based experiment to test *H1*, namely, air pollution increases variety-seeking. After controlling for age, education, income, and mood, the analysis revealed that individuals who were exposed to photos of polluted sites were more likely to engage in variety-seeking behavior when making purchase decisions. Study 1 revealed the main effect of air pollution on variety-seeking. Next, to test the underlying mechanism, we conducted Study 2.

### 3.3 Study 2: mediating effect of need for arousal

Study 2 aimed at testing *H2*, the mediating effect of need for arousal, and extending the product category by chocolate as the choice set. A similar procedure of Study 1 was implemented and we predicted that the need for arousal would mediate the effect of air pollution on variety-seeking.

#### 3.3.1 Participants and procedure

##### 3.3.1.1 Participants

A 2 (air pollution: high air pollution condition vs. low air pollution condition) between-factor design was used. Ninety-seven valid participants were recruited for RMB¥6.00 payment through Credamo, a frequently used website for gathering data in China; the participant characteristics were as follows: 37.11% were women, with M_age_ of 31.58 (SD = 8.41); 79.38% were unmarried; and participants' median income was between RMB¥10,000 and RMB¥15,000.

##### 3.3.1.2 Air pollution manipulation

We randomly assigned subjects to the high air pollution condition (*n* = 49) and low air pollution condition (*n* = 48). We used the same manipulation process as that used in Study 1. That is, participants in the high air pollution condition were presented with six photos taken on days with severe air pollution and were instructed to imagine currently living in that city. They were instructed to write in detail (at least 25 words) about living in that city. Participants in the low air pollution condition observed photos taken on a blue-sky day at the same sites as those in the high air pollution condition; the pictures were, therefore, similar except for the air pollution condition. The manipulation check of air quality was conducted by asking each group of participants to rate the air quality of the photo shown to them on a seven-point scale from 1 (indicating very bad) to 7 (indicating very good) (Liu et al., 2022).

##### 3.3.1.3 Measurements of need for arousal and variety-seeking

After the manipulation, we measured their need for arousal by assessing the extent to which they agreed or disagreed with the following statement: “I need much more arousal” on a seven-point scale (1 = strongly disagree, 7 = strongly agree) (Huang et al., 2019). Then, we asked the participants to imagine that they needed to choose three pieces of chocolate bars from six popular chocolate bars (Snickers Peanut Sandwich Chocolate, Leconte Nuts Cereal Chocolate, Hershey Cookies Dark Chocolate, Kinder Milk Hazelnut Wafer Chocolate, Dove Milk Chocolate, and Meiji Dark Chocolate) and buy them in any combination. The number of different brands they chose was used as a measurement of variety-seeking.

Finally, demographic information, including age, gender, city, and income, was collected.

#### 3.3.2 Results

##### 3.3.2.1 Manipulation check

A t-test was performed for the manipulation check, and the results indicate successful manipulation (M_low air pollution_= 6.54 vs. M_high air pollution_= 1.20, *t* = 48.30, *p* < 0.001).

##### 3.3.2.2 Mediating effect of need for arousal

**Figure 2:**
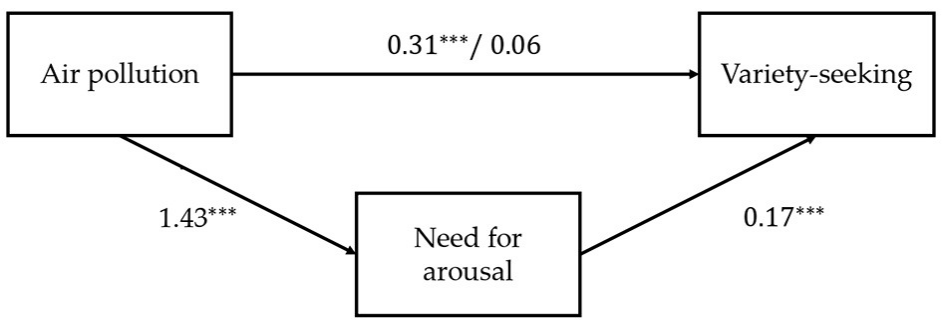
Mediating effect of the need for arousal. *p < 0.1, **p < 0.05, ***p < 0.01.

Next, we performed a series of linear regression analyses to test the mediating effect of the need for arousal. First, we regressed variety-seeking on air pollution and demographic control variables, namely age, gender, education, income, and city. We found a significant increasing effect of air pollution on variety-seeking (*b* = 0.31, *t* = 4.43, *p* < 0.001). Second, we regressed the need for arousal on air pollution and control variables, namely age, gender, education, income, and city. We found a significant positive coefficient of air pollution (*b* = 1.43, *t* = 5.22, *p* < 0.001). The results indicate that air pollution increased participants' need for arousal. Finally, we regressed variety-seeking on air pollution, the need for arousal, and the same control variables used in steps 1 and 2. The results reveal that the need for arousal significantly increased variety-seeking (*b* = 0.17, *t* = 6.30, *p* < 0.001), and the coefficient of air pollution was positive but non-significant (*b* = 0.06, *t* = 0.98, *p* = 0.33). Overall, we found a full mediating effect of the need for arousal, thus supporting *H2*. The model coefficients are shown in Figure 2.

#### 3.3.3 Discussion

In Study 2, we used a primed air pollution design to test *H2*, which is the mediating effect of the need for arousal on variety-seeking. The results confirm that there was a full mediating effect of the need for arousal, which provides insights into the mechanism and supports *H2*. Subsequently, we conducted Study 3 to test the moderation effect of visibility and how air pollution affected variety-seeking in a real-world setting, which would extend managerial implications and generalizability.

### 3.4 Study 3: natural experiment of actual purchase and moderation effect of visibility

Study 3 used a natural experiment with randomly chosen samples of actual purchases to test *H3*, namely, the moderation effect of visibility and the external validity. We predicted that the main effect would hold in the real world setting and visibility weaken the air pollution's positive effect on variety seeking.

#### 3.4.1 Data and methodology

##### 3.4.1.1 Data

We randomly selected 526 consumer transaction records from a cooperative chain-store fruit retailer based in Beijing during the period from 1 July 2018 to 31 December 2018. The cooperative fruit retailer is one of the largest retailers in Beijing and guarantees representativeness. The consumers originated from 20 different districts, with 96.95% of them residing in Beijing. All the transaction records of the consumers in any of the chain stores were collected. The transaction data included the purchased item, price, coupon, date, and store location information. We matched the transaction data to the nearest weather station using the store location, and we obtained the air quality index (AQI) and weather data (i.e., temperature, rain, visibility, air pressure, and humidity) at the time the transactions took place. The data were at the consumer–date level, resulting in a total of 290,400 observations; the descriptive statistics are shown in Table 1^3^. The maximum number of SKUs, which represent a distinct type of item for sale, purchased by consumers in a single day was 9, with an average of 0.01 SKU of fruits purchased per day, implying that consumers in our record purchased one SKU of fruits every 10 days on average. Considering that consumers may purchase elsewhere and that both heavy and light consumers exist, the data we observed are reasonable.

**Table 1:** Descriptive statistics.

	**Observations**	**Mean**	**SD**	**Median**	**Min**.	**Max**.
SKU	290,400	0.01	0.1	0	0	9
AQI > 300	287,228	0.04	0.2	0	0	1
Sales	290,400	1.91	17.79	0	0	5394.84
Coupon	290,400	0.01	0.28	0	0	49.91
Visibility	288,787	11.18	8.12	9.3	0.2	30
Temperature	288,787	14.36	11.17	16.4	−14.3	32.9
Rainfall	289,327	0.91	6.41	0	0	140.5
Air pressure	288,787	1010.77	10.14	1011	990	1043
Humidity	288,787	55.72	20.4	57	9	100

##### 3.4.1.2 Methodology

We used a fixed-effect model, similar to that used in the study of Liu et al. (2022), to analyze the impact of air pollution on consumers' variety-seeking behavior and the moderation effect of visibility. Model (1) was used for the main effect and model (2) was used for the moderation effect of visibility.


$$\begin{array}{l} \#\text{ of }{\text{SKU}}_{\text{it}}\text{= }\alpha *\text{if AQI}>{\text{300}}_{\text{it}}\text{+ }\beta_{\text{1}}*{\text{Sale}}_{\text{it}}\text{+ }\beta_{\text{2}}*{\text{Coupon}}_{\text{it}}\\ \text{ +}\beta_{\text{3}}*{\text{Weather}}_{\text{it}}\text{+ }{\text{c}}_{\text{i}}\text{+ }{\text{d}}_{\text{d}}\text{+ }\varepsilon_{\text{it}}\text{ Model }(\text{1}) \end{array}$$

$$\begin{array}{l} \;\#\text{ of }{\text{SKU}}_{\text{it}}\\ =\;\alpha *\text{if AQI}>{\text{300}}_{\text{it}}\text{+}\gamma *\text{if AQI}>{\text{300}}_{\text{it}}*{\text{Visibility}}_{\text{it}}\\ \;+\;\beta_{\text{1}}*{\text{Sale}}_{\text{it}}\text{+ }\beta_{\text{2}}*{\text{Coupon}}_{\text{it}}\text{+}\beta_{\text{3}}*{\text{Weather}}_{\text{it}}\\ \;+\;{\text{c}}_{\text{i}}\text{+ }{\text{d}}_{\text{d}}\text{+ }\varepsilon_{\text{it}}\text{ Model }(\text{2}) \end{array}$$

The variable *#of SKU*_*it*_ measures the amount of fruit SKUs purchased by *Consumer*_*i*_ on *date*_*t*_, which represents the level of variety-seeking. The AQI is a value between 0 and 500 and is the most widely used parameter for reporting air quality. The AQI is calculated based on a complex weighted average of the concentrations of various pollutants in the air, such as SO_2_, NO_2_, PM_10_, PM_2.5_, O_3_, and CO. The higher the AQI, the greater is the level of air pollution and the greater is the health concern. An AQI <150 indicates good air quality, causing no major health concerns, whereas an AQI >300 represents hazardous air quality, which adversely affects most people.^4^ The variable *if AQI*>300_*it*_ indicates whether the AQI at the consumer's location on *date*_*t*_ was above 300, serving as a measure of the severity of air pollution. *Sale*_*it*_ represents the total expenditure on fruit categories, *Coupon*_*it*_ represents the discount amount, and *Weather*_*it*_represents the local weather variables, including visibility, temperature, rainfall, air pressure, and humidity. *c*_*i*_ represents consumer fixed effects, *d*_*t*_ represents date fixed effects, and ε_*it*_ is a disturbance term. For the moderation effect, we added the product of *if*_*AQI*_ > 300_*it*_ and *Visibility*_*it*_ in Model (1) to form Model (2) as a test.

#### 3.4.2 Results

**Figure 3:**
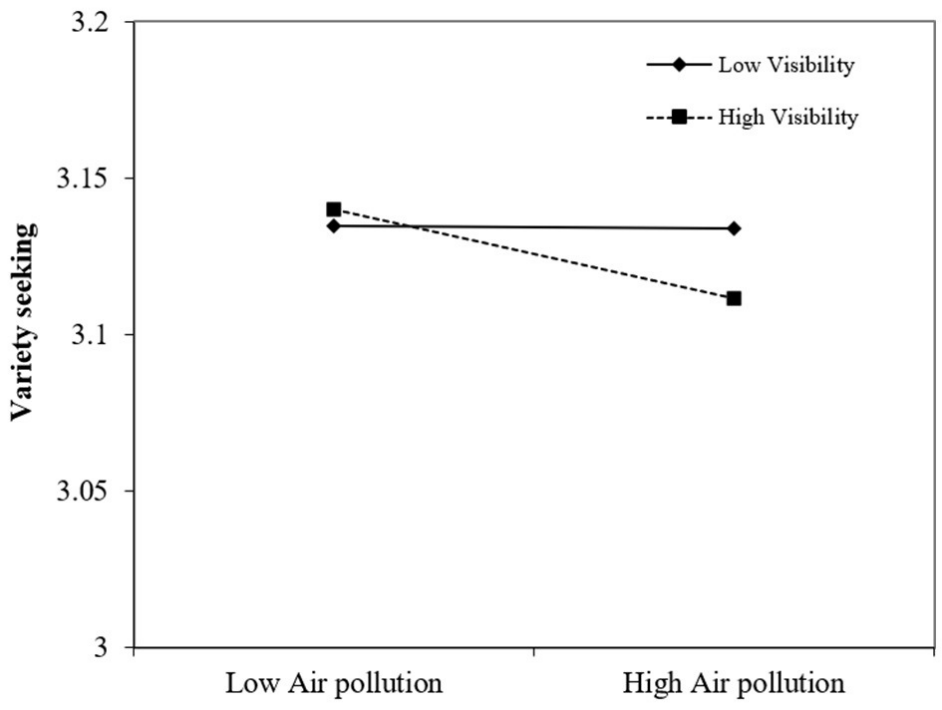
Moderation role of visibility.

The results are presented in Table 2, where column (1) shows the results of Model (1) for the main effect and column (2) shows the results of Model (2) for the moderation effect of visibility. Column (1) shows that, when the AQI was above 300, indicating hazardous air quality, consumers tended to purchase an additional 0.00229 SKU compared to when the AQI was below 300. Considering that the average number of SKUs purchased was 0.01, the effect of air pollution on variety-seeking was equivalent to a 22.9% increase in the average number of SKUs purchased. In column (2), we found a significant negative moderation effect of visibility after adding the interaction term of visibility. A plot of the interaction effect is shown in Figure 3. The results confirm the external validity of our findings and support *H3*, namely, the effect of air pollution on variety-seeking was moderated by visibility. Thus, these results strengthened the generality of our findings and deepened our understanding.

**Table 2:** Effect of air pollution on variety-seeking.

	**(1)**	**(2)**
	**Variety-seeking**	
Sale	0.000919^***^ (0.000)	0.000919^***^ (0.000)
AQI > 300	0.00229^*^ (0.053)	0.00468^**^ (0.013)
Coupon	0.220^***^ (0.000)	0.220^***^ (0.000)
Visibility	0.000324 (0.222)	0.000331 (0.216)
Temperature	−0.000120 (0.875)	−0.000202 (0.800)
Rainfall	−0.0000631 (0.321)	−0.0000631 (0.319)
Air pressure	0.00106 (0.470)	0.00114 (0.434)
Humidity	0.0000751 (0.573)	0.000294 (0.394)
AQI > 300^*^Visibility		−0.00172^**^ (0.011)
Observations	285,630	285,630

The p-values are shown in parentheses, and the robust SDs are aggregated at the individual level. ^*^p <0.1, ^**^p <0.05, ^***^p <0.01.

## 4 Overall discussion

In this study, we explored the impact of air pollution on consumers' variety-seeking behavior, its mechanisms, and boundary conditions through two online experiments and one natural experiment. Study 1 provided the initial evidence that air pollution has a significant increasing effect on consumers' variety-seeking behavior. Study 2 provided further support for the main effect, and the results indicated the mediating effect of the need for arousal. Finally, through a natural experiment, Study 3 demonstrated the external validity and confirmed the moderation effect of visibility, as well as provided insights for managers.

### 4.1 Theoretical contributions

The effects of air pollution on physical health, mental health, and cognitive ability and their secondary effects have been widely investigated. This research extends the literature by investigating how air pollution affects human behavior from the perspective of visual effects. We found that an air pollution-induced achromatic environment increases the need for arousal and that variety-seeking is a response for fulfilling the need for arousal.

Our research contributes to a better understanding of the effects of air pollution on variety-seeking. The results of the study revealed that the need for arousal mediates the relationship between air pollution and variety-seeking and that visibility weakens this positive relationship. Thus, the findings enrich our understanding of the effects of air pollution on human behavior.

### 4.2 Practical contributions

The findings of this research offer practical implications for product category managers. First, when air pollution is at a high level, managers can increase the variety of products to enhance sales. This is based on our findings that consumers prefer more varied products when the environment is subject to air pollution and that managers can better meet consumer demand by providing a greater variety of products. Second, advertisements can emphasize the arousal-fulfilling feature of products when people are exposed to air pollution. Based on our finding that consumers exhibit greater arousal-seeking behavior when the environment is subject to air pollution, it is reasonable to predict that arousal-fulfilling features can grab consumers' attention and that advertisements focusing on such arousal-fulfilling features would be more effective. Third, visibility should be taken into account when implementing the two abovementioned recommendations. This research revealed that visibility negatively moderates the positive effect of air pollution on variety-seeking through the need for arousal. When visibility is low and high, air pollution-induced effects are partially eliminated, making the above mentioned measures ineffective.

Global environmental changes have become one of the major challenges. To address this challenge, countries and companies should be able to anticipate how different environmental factors affect human behaviors. The findings of this research can also help government and policymakers gain a better understanding and aid them in establishing environment-related policies.

### 4.3 Limitations and future directions

This research has several limitations. First, the findings are limited to China and the product categories we tested. Participants in Study 1 and Study 2 were recruited from China, and most of the consumers for Study 3 were located in Beijing, where the air pollution conditions are moderately severe. Whether our findings hold in other regions and countries needs further research. In addition to the generality of the location, our results are limited to beverage, chocolate, and fruit categories, which we tested in Study 1, Study 2, and Study 3, respectively.

Second, how air pollution affects people is a complex phenomenon, and there might be other underlying mechanisms. Previous research has shown that physical confinement increases variety-seeking through the need for freedom (Levav and Zhu, 2009). As a health-threatening environmental factor, air pollution inhibits people from going outside (Zivin et al., 2009). Whether these information channels contribute to our findings is worthy of future research by employing the Mindsponge Theory and the Bayesian Mindsponge Framework (Vuong et al., 2022b; Vuong, 2023), which are effective information processing tools.

Third, how air pollution-induced need for arousal affects other stimulus-seeking behaviors is worthy of future investigation. The need for arousal affects a wide range of behaviors, including risk taking behavior (Guiso et al., 2018), mood congruent behavior (Di Muro and Murray, 2012), and product evaluation (Noseworthy et al., 2014). Future research should explore the effects of air pollution-induced need for arousal on various other behaviors as air pollution also involves other mechanisms.

## Data availability statement

The raw data supporting the conclusions of this article will be made available by the authors, without undue reservation.

## Ethics statement

The studies involving humans were approved by the Minjiang University. The studies were conducted in accordance with the local legislation and institutional requirements. The participants provided their written informed consent to participate in this study.

## Author contributions

HZ: Conceptualization, Formal analysis, Funding acquisition, Methodology, Project administration, Writing – review & editing. GH: Investigation, Writing – original draft. PL: Funding acquisition, Writing – review & editing. XC: Writing – review & editing, Funding acquisition. WL: Funding acquisition, Writing – review & editing.

## Footnotes

^1^The standards for air quality stipulated by the WHO are an annual average PM_10_ level of less than 20 μg/m3 and an annual average PM_2.5_ level of less than 10 μg/m3. Source: https://www.who.int/news-room/fact-sheets/detail/ambient-(outdoor)-air-quality-and-health.

^2^Source: https://www.who.int/news/item/12-05-2016-air-pollution-levels-rising-in-many-of-the-world-s-poorest-cities.

^3^There are missing observations for weather data, so the number of observations for weather variables may be less than 290,400.

^4^Source: https://www.airnow.gov/aqi-and-health/.
