# Supplementary material for: Visual effect of air pollution on the need for arousal and variety-seeking behavior
Source: Front Psychol. 2024 May 23;15:1342267. doi: 10.3389/fpsyg.2024.1342267 (PMC11154011; doi:10.3389/fpsyg.2024.1342267)
Supplement: Supplementary file 1 [file Table_1.docx]

Supplementary Material

# Demographic statics of Study 1

Table 1. Demographic Statistics of Study 1.

| Items | Classification | Frequency | Percentage (%) |
| --- | --- | --- | --- |
|  | Under 20 | 8 | 8% |
| Age | 21–30 | 43 | 43% |
|  | 31–40 | 41 | 41% |
|  | 41 or older | 8 | 8% |
|  | Senior high school and equivalent or below | 2 | 2% |
| Education | Junior college | 4 | 4% |
|  | Bachelor’s degree | 78 | 78% |
|  | Master’s degree | 16 | 16% |
|  | Less than 2000 | 9 | 9% |
|  | 2000–5000 | 12 | 12% |
| Income | 5000–8000 | 14 | 14% |
|  | 8000–10,000 | 18 | 18% |
|  | 10,000–15,000 | 32 | 32% |
|  | 15,000 or more | 15 | 15% |

# Model used in Study 1

$$\# of SKU= c+ \alpha* if air pollution group {+ \beta}_{1}*age + \beta_{2}*income level + \beta_{3}*education level +\beta_{4}*\mathrm{mood}+ \varepsilon$$

$\# of SKU$ was the number of unique beverages chosen, which represented level of variety-seeking. If air pollution group was an indication of manipulation, it equaled one when participants were in the high air pollution group and equaled zero when participants were in the low air pollution group. Age, income level and education level represented the literal meanings. $\varepsilon$was the error term and c was the intercept.
