# Supplementary material for: Visual effect of air pollution on the need for arousal and variety-seeking behavior
Source: Front Psychol. 2024 May 23;15:1342267. doi: 10.3389/fpsyg.2024.1342267 (PMC11154011; doi:10.3389/fpsyg.2024.1342267)
Supplement: Supplementary file 2 [file Table_2.docx]

Supplementary Material

# Demographic statics of Study 2

Table 2. Demographic Statistics of Study 2.

| Items | Classification | Frequency | Percentage (%) |
| --- | --- | --- | --- |
|  | Under 20 | 7 | 7.22% |
| Age | 21–30 | 27 | 27.84% |
|  | 31–40 | 55 | 56.7% |
|  | 41 or older | 8 | 8.25% |
|  | Senior high school and equivalent or below | 1 | 1.03% |
| Education | Junior college | 9 | 9.28% |
|  | Bachelor’s degree | 77 | 79.38% |
|  | Master’s degree | 10 | 10.31% |
|  | Less than 2000 | 6 | 6.19% |
|  | 2000–5000 | 15 | 15.46% |
| Income | 5000–8000 | 15 | 15.46% |
|  | 8000–10,000 | 12 | 12.37% |
|  | 10,000–15,000 | 13 | 13.4% |
|  | 15,000 or more | 36 | 37.11% |

# Models used in Study 2

A serial of ordinary least square linear regressions were implemented in Study 2. Models used in step1, step 2 and step 3 were listed below.

Step1: $\#of SKU=c+\alpha*if air pollution group+ \beta_{1}*age+\beta_{2}*income level+\beta_{3}*education level+ \beta_{4}*city+ \varepsilon$

Step2: $need for arousal=c+\alpha*if air pollution group+ \beta_{1}*age+\beta_{2}*income level+\beta_{3}*education level+ \beta_{4}*city+\varepsilon$

Step3: $\#of SKU=c+\alpha*if air pollution group+\gamma* need for arousal+ \beta_{1}*age+\beta_{2}*income level+\beta_{3}*education level+ \beta_{4}*city+ \varepsilon$

$\#of SKU$ was the number of unique chocolates chosen, which represented level of variety-seeking. If air pollution group was an indication of manipulation, it equaled one when participants were in the high air pollution group and equaled zero when participants were in the low air pollution group. Need for arousal, age, income level, education level and city represented the measurements of need for arousal, age, income level, education level and city of the participants. $\varepsilon$was the error term and c was the intercept.
