# Supplementary material for: Visual effect of air pollution on the need for arousal and variety-seeking behavior
Source: Front Psychol. 2024 May 23;15:1342267. doi: 10.3389/fpsyg.2024.1342267 (PMC11154011; doi:10.3389/fpsyg.2024.1342267)
Supplement: Supplementary file 3 [file Table_3.docx]

Supplementary Material

# Experiment materials

# Air pollution manipulation (Study 1& 2)

*Instructions:* Please imagine the air quality of the city where you are currently living as shown in the photos, and you are breathing, working and studying under such air quality. Then describe how you would feel and experience living in this environment for one day in detail. For example, how would you feel when going outside? (minimum of 25 words).

| Photo used in air pollution condition | Photo used in blue-sky condition |
| --- | --- |
| 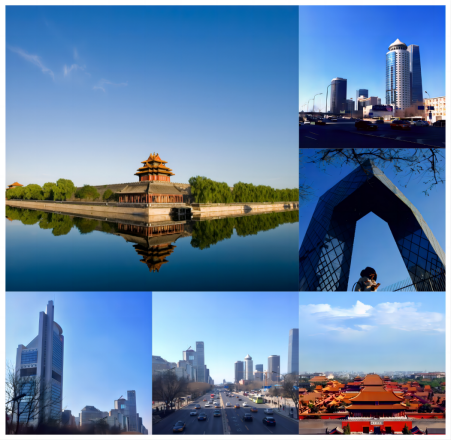 | 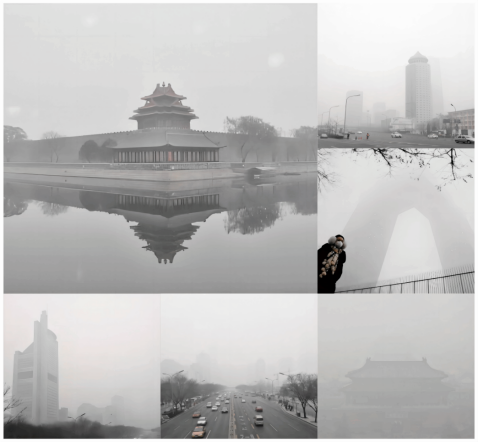 |

Liu, J., Zou, P., and Ma, Y. (2022). The Effect of Air Pollution on Food Preferences. Journal of the Academy of Marketing Science 50, 410–423. doi: 10.1007/s11747-021-00809-8.

# Manipulation check (Study 1& 2)

*Instructions*：Participants in each group were asked the following questions.

How would you rate the air quality of the city on the day the photo was taken?

| 1 | 2 | 3 | 4 | 5 | | 6 | | 7 | |
| --- | --- | --- | --- | --- | --- | --- | --- | --- | --- |
| very bad |  |  |  |  |  | | very good | |  |

Liu, J., Zou, P., and Ma, Y. (2022). The Effect of Air Pollution on Food Preferences. *Journal of the Academy of Marketing Science* 50, 410–423. doi: 10.1007/s11747-021-00809-8.

# Variety seeking (Study 1)

*Instructions:* Please choose your favorite 3 bottles of beverages from the following 6 types. You can choose 3 bottles of any combination of the beverages and the total number should be 3. For example, you can choose 3 bottles of Coca-Cola or you can choose 1 bottle of Sprite and 2 bottles of Nongfu Spring, for a total of 3 bottles. How would you choose?


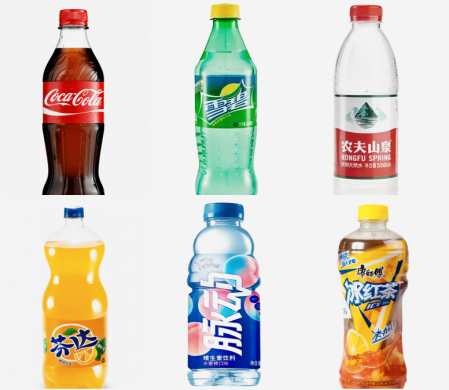


# Mood (Study 1)

How are you feeling right now?

| 1 | 2 | 3 | 4 | 5 | | 6 | | 7 | |
| --- | --- | --- | --- | --- | --- | --- | --- | --- | --- |
| very bad |  |  |  |  |  | | very good | |  |

Ding, Y., Zhong, J., Guo, G., and Chen, F. (2021). The impact of reduced visibility caused by air pollution on construal level. *Psychology & Marketing* 38, 129–141. doi: 10.1002/mar.21427.

# Need for Arousal (Study 2)

*Instructions:* Please rate the statement from 1 (strongly disagree) to 9 (strongly agree) according to what you feel right now.

I need much more arousal.

Huang, Z. (Tak), Liang, Y. (Sky), Weinberg, C. B., and Gorn, G. J. (2019). The Sleepy Consumer and Variety Seeking. *Journal of Marketing Research* 56, 179–196. doi: 10.1177/0022243718811334.

# Variety seeking (Study 2)

*Instructions:* Suppose that you need to buy 3 pieces of chocolate and please choose 3 pieces from the following 6 types of chocolate. You can choose 0-3 pieces of each flavor and the total number of pieces should be 3. For example, you can choose 3 pieces of Snickers Peanut Sandwich Chocolate, or you can choose 1 piece of Dove Milk Chocolate and 2 pieces of Hershey Cookies Dark Chocolate respectively, all of which equal 3 pieces in total. How would you choose?


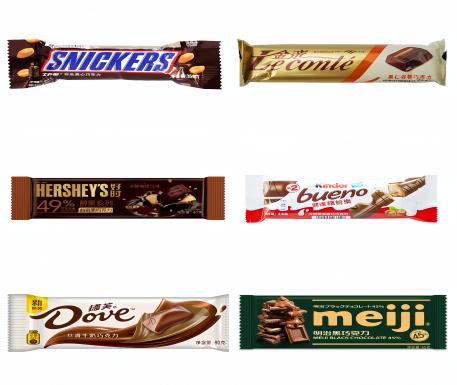


Liu, J., Zou, P., and Ma, Y. (2022). The Effect of Air Pollution on Food Preferences. *Journal of the Academy of Marketing Science* 50, 410–423. doi: 10.1007/s11747-021-00809-8.
